# Dialysis Preparation of Smart Redox and Acidity Dual Responsive Tea Polyphenol Functionalized Calcium Phosphate Nanospheres as Anticancer Drug Carriers

**DOI:** 10.3390/molecules25051221

**Published:** 2020-03-09

**Authors:** Xiuli Ren, Peng Zhang, Zhenhua Chen

**Affiliations:** College of Basic Science, Jinzhou Medical University, Jinzhou 121001, China; rxlrenxiuli@163.com (X.R.); zxc2129563@163.com (P.Z.)

**Keywords:** calcium phosphate, nanospheres, drug delivery, pH-responsive, doxorubicin

## Abstract

Large-scale preparation of biocompatible drug delivery systems with targeted recognition and controlled release properties has always been attractive. However, this strategy has been constrained by a lot of design challenges, such as complicated steps and premature drug release. Herein, in this paper, we address these problems by a facile in situ mineralization method, which synthesizes biodegradable tea polyphenol coated monodisperse calcium phosphate nanospheres using for targeted and controlled delivery of doxorubicin. Dialysis diffusion method was used to control ion release to form mineralized nanospheres. The polyphenol coatings and calcium phosphate used in this work could be biodegraded by intracellular glutathione and acidic microenvironment, respectively, resulting the release of encapsulated drug. According to confocal fluorescence microscopy, and cytotoxicity experiments, the prepared tea polyphenol functionalized, doxorubicin loaded calcium phosphate nanospheres were confirmed to have highly efficient internalization and obvious cell killing effect on target tumor cells, but not normal cells. Our results suggest that these tea polyphenols functionalized calcium phosphate nanospheres are promising vehicles for controlled release of an anticancer drug in cancer therapy.

## 1. Introduction

The development of efficient targeted and multi-responsive drug delivery systems (DDS) used for controlled release of anticancer drug has attracted much attention [1,2]. Various biocompatible nanoparticles, for example, polymers, inorganic nanospheres and capsules, metal organic framework, and mesoporous silica, have been used as nano DDS [1,2,3,4,5]. These studies have proposed a promising strategy, i.e., design DDS with various stimuli like temperature, ultrasound, enzymes, light, reactive oxygen species, pH, and redox agents, to overcome the drawbacks of conventional drugs, such as high toxicity and poor specificity in tumor [6,7,8,9,10]. Among those stimuli, the pH and redox agents are mostly used in DDS, probably because physiological microenvironments cover a wide pH range and the redox process is very common in human body [1,2]. Compared with normal tissues, most tumors have a weak acidic microenvironment and exhibit elevated concentration of glutathione (GSH) [1,2]. Hence, the pH and GSH gradients are promising triggers for multi-responsive DDS. Large-scale preparation of biocompatible drug delivery systems with targeted recognition and controlled release properties has always been attractive. However, this strategy has been constrained by a lot of design challenges, such as complicated steps in design and preparation of GSH-responsive substrates, and premature drug release.

**Figure 1:**
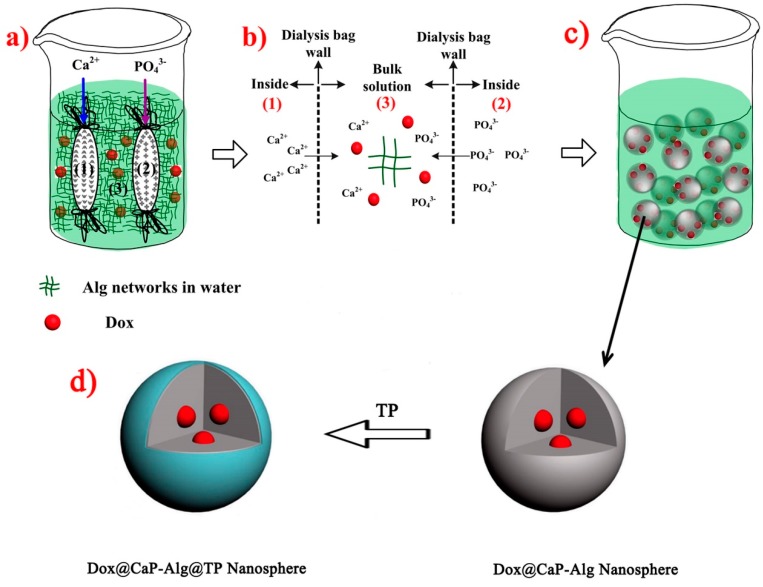
Schematic preparation process of Dox@CaP-Alg@TP (DCAT) nanospheres. (**a**) reaction system, (**b**) detail illustration of Ca^2+^ and PO_4_^3−^ gradually diffuse into the Alg networks, (**c**) illustration of formed CaP nanoparticles, (**d**) TP function process of CaP NPs to form DCAT NSs.

**Figure 2:**
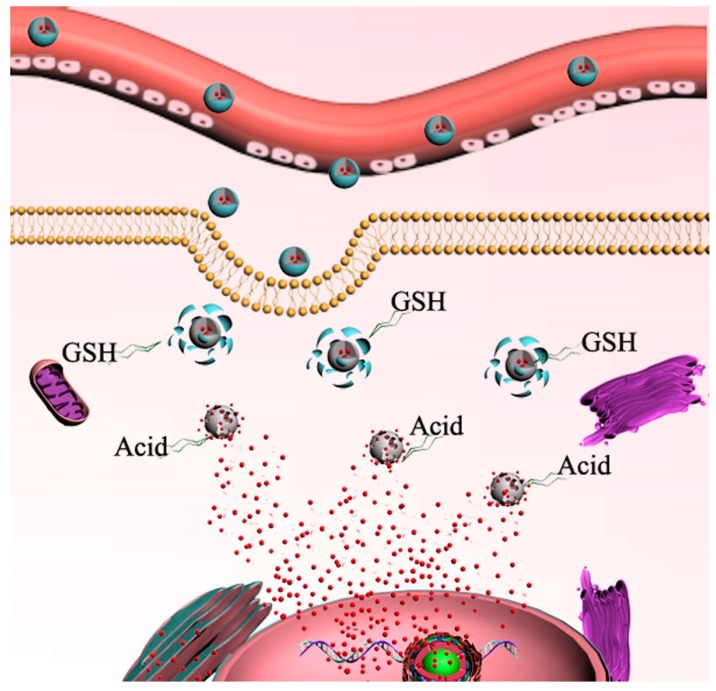
The illustration of DCAT nanospheres used as promising vehicles for controlled release of anticancer drug. Polyphenol coatings and calcium phosphate could be biodegraded by intracellular glutathione and acidic microenvironment, respectively, resulting the release of encapsulated drug.

Herein, in this paper, as illustrated in Figure 1, we address these problems by a facile in situ mineralization method [11], which synthesizes biodegradable tea polyphenol coated monodisperse calcium phosphate nanospheres for targeted and controlled delivery of doxorubicin. This proposal is based on our previous research work and reasoning as follows: our former work and the following outstanding research work from other groups have proved that polyphenol structures can be degraded at high GSH concentrations [12,13,14]. Various of inorganic nanomaterials, such as iron oxides, carbon nanotubes, noble metals, silica, calcium phosphates, and quantum dots have contributed greatly to many biomedical breakthroughs [15]. Among them, calcium phosphates (CaP) have no concerns about their inherent cell toxicity because they are the inorganic component of biological hard tissues, i.e., bone and teeth [14,16,17]. Especially, CaP can degrade in acidic environments [17,18,19,20,21]. Therefore, as shown in Figure 2, the polyphenol coatings and calcium phosphate used in this work could be biodegraded by intracellular glutathione and acidic microenvironment, respectively, resulting the release of encapsulated drug. These tea polyphenols functionalized calcium phosphate nanospheres are promising vehicles for controlled release of anticancer drug in cancer therapy.

## 2. Results and Discussion

**Figure 3:**
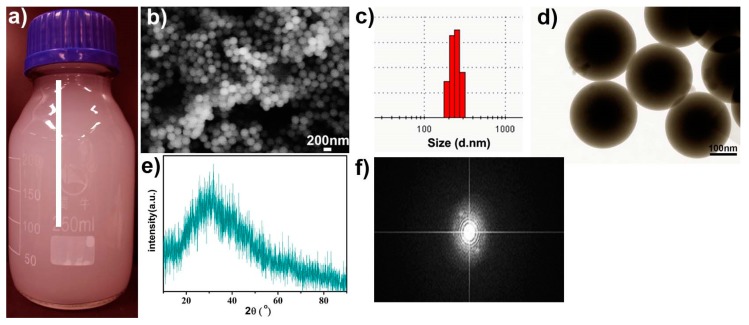
Photograph (**a**), scanning electron microscopy (SEM) (**b**), dynamic laser light scattering (DLS) (**c**) and transmission electron microscopy (TEM) (**d**) images, and X-ray diffraction (XRD) (**e**), and Fast Fourier Transformation (FFT) (**f**) images of the prepared DCAT nanospheres.

The photograph in Figure 3a clearly shows that the prepared products are well dispersed in water. The SEM image in Figure 3b reveals that the obtained products are large amount of monodisperse spheres with an average size of 260 nm in diameter. DLS size distribution result (Figure 3c) further indicated that the prepared nanospheres have a narrow size range. The TEM image in Figure 3d indicates that each of the prepared uniformed DCAT nanospheres has a dark core and lighter outer layer. The amorphous structure of the nanospheres are confirmed by the non-diffraction pattern (Figure 3e) and the diffraction pattern (Figure 3f) in the FFT (Fast Fourier Transformation) image.

**Figure 4:**
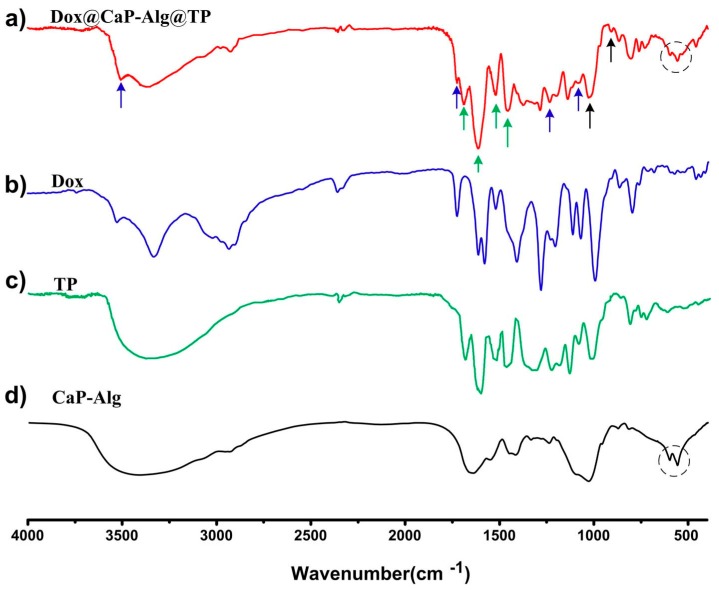
Fourier transform infrared spectroscopy (FTIRI spectra of the samples: (**a**) DCAT nanospheres; (**b**) Dox; (**c**) tea polyphenols (TP), and (**d**) CaP-Alg.

Figure 4a–d are the infrared spectra of DCAT nanospheres, Dox, TP, and CaP-Alg. The cycles marked in Figure 4a,d indicate the characteristic absorptions of CaP. The two bands ascribed to PO_4_^3−^ have shifted form (567 cm^−1^, 609 cm^−1^; Figure 4d) to (567 cm^−1^, 609 cm^−1^; Figure 4a). This might suggest that, in the hybrid materials, TP or Dox could have interactions with CaP part. As shown in Figure 4a (black arrows), the absorption peak at 1425 and 1384 cm^−1^ result from the cross-linking of COO^−^ (Alg) with Ca^2+^ (CaP). This may suggest that Alg has been incorporated into CaP. The absorptions at 3425, 1729, 1286, and 1007 cm^−1^ are owing to Dox (Figure 4a, indicated by blue arrows). Absorptions at 1700, 1637, 1519, and 1450 cm^−1^ may originate from the TP (Figure 4a, indicated by green arrows). The peaks of OH^−^ and PO_4_^3−^ are 1030 and 960 cm^−1^, respectively. These data indicate that Dox molecules were successfully packaged into DCAT NSs.

**Figure 5:**
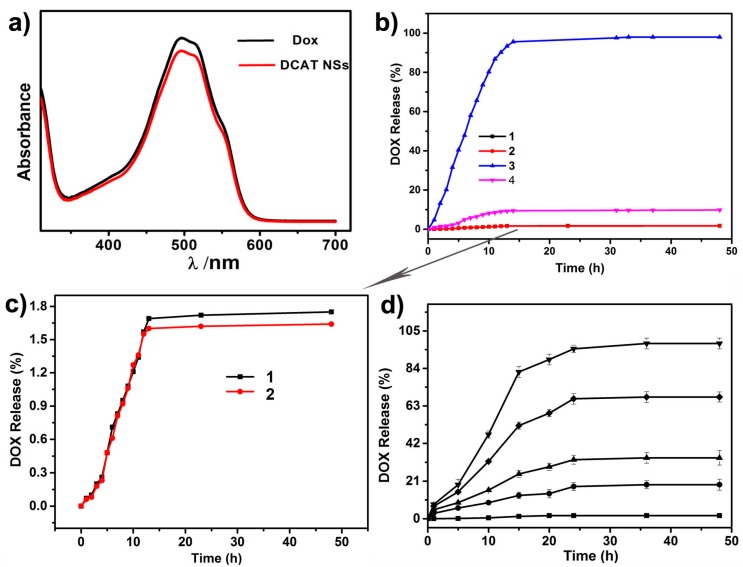
(**a**) UV-vis spectra of Dox and DCAT NSs; (**b**) release profiles of Dox from DCAT NSs at pH = 7.4 (1, 2) and 5.0 (3, 4), in the absence (2, 4) and presence (1, 3) of 4.2 mM GSH; (**c**) the magnified profiles 1 and 2 in (**b**); (**d**) GSH-dependent Dox release from DCATP NSs in the presence of different glutathione concentration at pH = 5.0.

The UV-vis spectrum of DCAT NSs resembles Dox’s, it has a maximum absorbance at about 480 nm which is the characteristic absorption peak of Dox (Figure 5a). Figure S1 show the calibration curve of Dox determined by taking absorbance versus Dox concentration between 0 and 1 × 10^−4^ mol·L^−1^ as parameters. As shown in Figure 5b, the acidic environment significantly accelerated the release of Dox. At pH = 7.4 (line 1,2), the maximum release of Dox from DCAT NSs is less than 2% in decades hours. Figure 5c further revealed that those drugs were released during the first few hours. This may be due to the release of a small amount of adsorbed drug. And there is no significant increase in release over the next tens of hours. However, while pH was adjusted to 5.0 (line 3,4; Figure 5b), the cumulative release amount is highly increased than those of measured at pH 7.4. Comparing the release curves 3 and 4 of Figure 5b carefully, it can also be found that in the absence of GSH, there is only a maximum release of about 10%; in the presence of GSH, there is a maximum release of 98%. Detailly GSH concentration depended release trend was present in Figure 5d. At pH = 5.0, while the concentration of GSH is increased, the increase of drug release is obvious. The synergistic effect of GSH and pH to control the release of Dox from nanoparticles will be explained in detail in the data of subsequent cell experiments. The CaP portion of the hybrid material corresponds to the acid response, and the TP portion corresponds to the GSH response. The initial state of TP might be oligo/polymeric TP before release, GSH could destruct the oligo/polymeric TP structure to cause dug release. Oligo/polymeric TP and GSH reduce release have been demonstrated in our former work focus on TP based nanomaterials [12].

**Figure 6:**
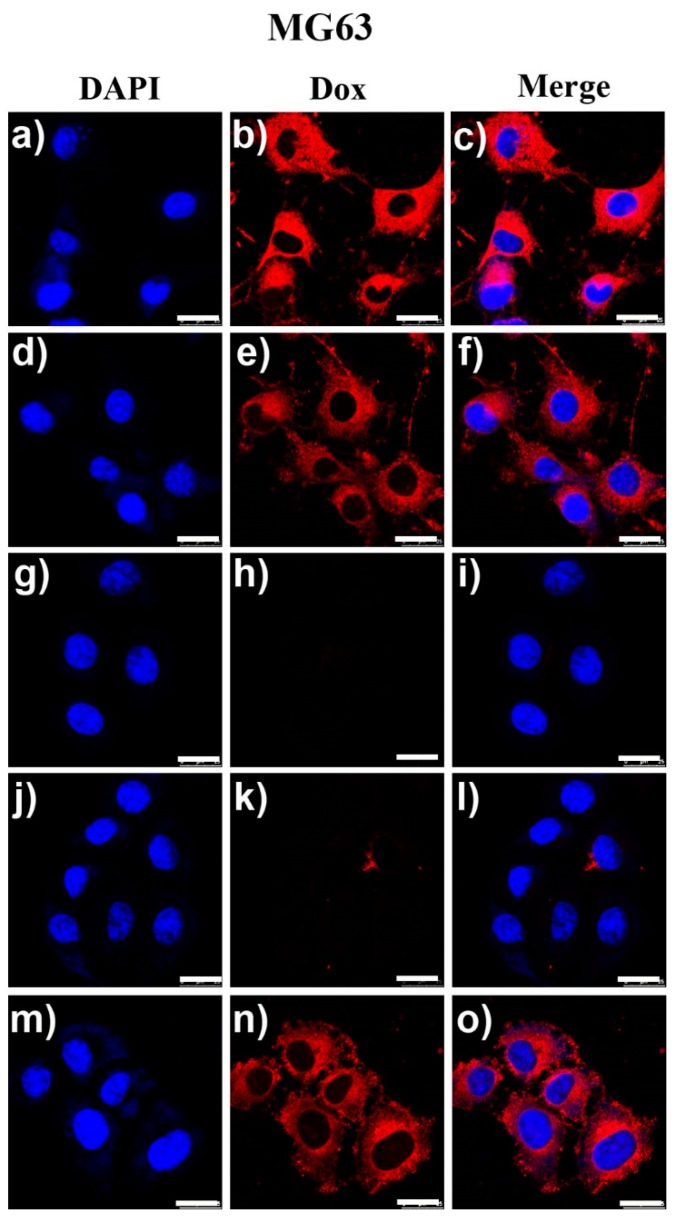
MG63 cells cultured in different conditions for 24 h: (**a**–**f**) with free Dox (1 μg/mL), (**g**–**i**) blank control group, and (**j**–**o**) with DCAT NSs (concentration of Dox = 1 μg/mL). Cells in (**a**–**c**) and (**j**–**i**) were cultured at pH = 7.4; (**d**–**f**) and (**m**–**o**) were at pH = 5.0.

**Figure 7:**
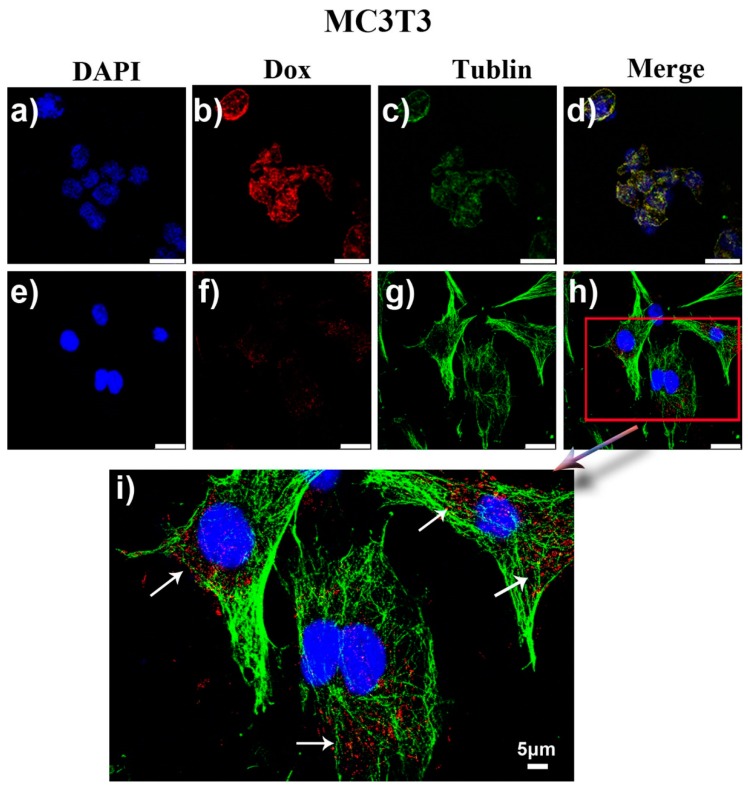
MC3T3 cells cultured in different conditions for 24 h: (**a**–**d**) with free Dox (1 μg/mL), (**e**–**h**) with DCAT NSs (concentration of Dox = 1 μg/mL); (**i**) magnified image of (**h**). The incubated pH is 7.4.

The above results from Figure 3, Figure 4 and Figure 5 proved that smart redox (GSH) and acidity dual responsive, tea polyphenol functionalized calcium phosphate nanospheres using as doxorubicin carriers have been prepared. The preparation strategy is inspired by the reported works about DDS focus on pH responsive [11] and GSH responsive [12,14]. Though the pH-folic acid, and GSH-enzyme dual responsive DDS have been reported [9,13], the obtained DCAT NSs further shed a light on pH-GSH dual responsive DDS. Figure 5 shows the release data of DCAT NSs. In order to further study the release mechanism, Figure 6 and Figure 7 show the results of co-culture of the DCAT NSs with tumor cells (MG63) and normal cells (MC3T3).

The results of MG63 cells co-cultured with free Dox and DCAT NSs were shown in Figure 6. Comparing the images in groups Figure 6a–c and Figure 6d–f, there were no difference in the red fluorescence intensity of doxorubicin in the two groups. Such result suggests that the change in pH did not significantly affect the doxorubicin intervention in MG63 cells. Figure 6g–i revealed the absence of red fluorescence from Dox in the blank control group. At pH = 7.4 (Figure 6j–i), owing to the tumor cells’ high GSH concentration (about 10 mM), like illustrated in Figure 1 and Figure 2, TP shells of those DCAT NSs would been broken and reduced to the original compound under the action of GSH as a reducing agent. Thus, a small amount of Dox would be released and a few Dox’s red fluorescence would be found in Figure 6k–l. Subsequently, DCAT NSs might be taken up by endocytosis and acidic environment there would further lyse the mineral part (calcium phosphate) to release the most part of encapsulated Dox very gradually. It is really hard to record this long release process by CLSM. However, after set the culture pH to 5.0, as shown in Figure 6m–o, strong red fluorescence from Dox was found in those CLSM images, which suggest that the synergistic effect of acidity and GSH can effectively promote Dox release from DCAT NSs.

**Figure 8:**
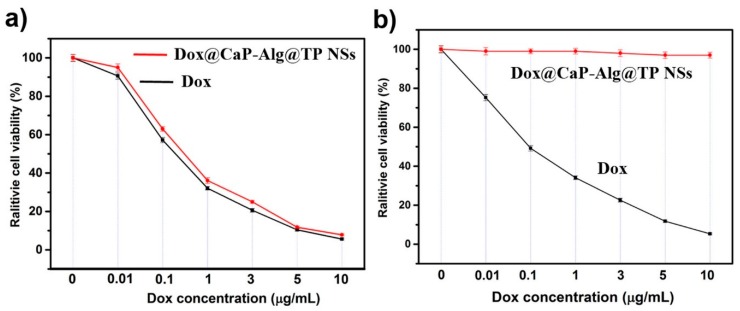
Cell viability results of cells cultured with DCAT NSs and free Dox under various concentrations: (**a**) MG63 cells and (**b**) MC3T3 cells.

In addition to osteosarcoma cells (MG63), we also investigated the differences between co-culture of Dox and DCAT NSs with pre-osteoblasts (MC3T3). When MC3T3 cells were incubated with Dox, Figure 7a–d reveal that the nucleus of MC3T3 cells appeared voids caused by lysis, the cytoskeleton was seriously atrophied, and the cells showed a trend of population apoptosis. These phenomena reveal the cytotoxicity of Dox on MC3T3 cells. At the same time, the necessity of using targeting carriers to reduce toxic side effects when using doxorubicin drugs was also verified. Unlike free Dox, the images (Figure 7e–h) of co-culture of DCAT NSs and MC3T3 cells showed that the cell morphology and cytoskeleton were still intact. The further enlarged image (Figure 7i) shows that although there are many DCAT NSs involved in the cells, the drug did not explode, because they still maintained a relatively dispersed nanospheres of fluorescent spots (indicated by arrows in Figure 7i). We speculate that this phenomenon is mainly due to the fact that MC3T3 cells’ intracellular lysosome has an acidic environment like MG63, but their GSH concentration is low, which is not enough to cause rapid degradation of the TP shell. Thus, the acidic environment is difficult to effectively destroy the inner layer of calcium phosphate (drug coating). Therefore, it is difficult for Dox to be effectively released, and MC3T3 cells can maintain intact morphology. The statistical results of cell viability results (Figure 8) further validate the conclusions in Figure 6 and Figure 7 that DCAT NSs have tumor cell killing effects similar to Dox, however, the side effects of toxicities in normal cells are much lower than Dox. The above results illustrate the efficiency and safety of this multiple response drug delivery carrier.

## 3. Materials and Methods

### 3.1. Materials

Sodium alginate (AR, 98%), CaCl_2_ (AR, 96%), NH_4_H_2_PO_4_ (AR, 98%), NH_3_·H_2_O (AR, 25%–28%), dimethyl sulfoxide (DMSO), dialysis tubing (pore size, 12,000 Da MWCO) and glutathione (GSH) were sourced from Sigma Chemical Co. (St Louis, MO, USA). Tea polyphenol (purity ≥ 98 wt.%, Wuxi Taiyo Green Power Co. Ltd., Jiangsu, China; chemical formula showed in Figure S2). MG63 and MC3T3 cells were purchased from the Institute of Basic Medical Science, Chinese Academy of Medical Sciences (Beijing, China). Doxorubicin Hydrochloride (Dox, wt.% > 98%, Beijing HuaFeng United Technology CO., Ltd., Beijing, China; chemical formula showed in Figure S3). Triply distilled deionized water was used during all the applications.

### 3.2. Characterization

Morphology of the products was investigated by scanning electron microscopy (SEM, Hitachi, S4800, Tokyo, Japan), and transmission electron microscopy (TEM, Tecnai G2 F20 S-TWIN, Hillsboro, OR, USA). The crystallographic structure of the products was measured by an X-ray diffraction (XRD, SHIMADZU, Kyoto, Japan). The size distribution of the products was characterized by dynamic laser light scattering (DLS, Malvern, Nano ZS90, Worcestershire, UK). The composition of the products was confirmed by Fourier transform infrared spectroscopy (FTIR, SHIMADZU, Kyoto, Japan) with the KBr disk method. UV data was recorded by UV/Vis spectrophotometer (U3010, Hitachi). The confocal laser scanning microscopy (CLSM, Leica TSC SP5 confocal unit, Buffalo Grove, IL, USA) was used to the cell uptake behavior of the products.

### 3.3. Preparation of Tea Polyphenol Functionalized Calcium Phosphate Nanospheres

As illustrated in Figure 1, the preparation of tea polyphenol functionalized calcium phosphate nanospheres (Dox@CaP-Alg@TP NSs, DCAT NSs) used dialysis diffusion method. Firstly, the mixed solution of sodium alginate (Alg) and Dox was placed in a beaker. Then, solutions of CaCl_2_ (0.05 mol/L, 20 mL) and the NH_4_H_2_PO_4_ (0.03 mol/L, 20 mL) (Ca/P = 1.67) packed in two separate dialysate tubes were put into the beaker mentioned above. After that, put the reaction system on experiment table at room temperature. In the first 3 h, pH was adjusted to 10.5 by NH_3_·H_2_O throughout the process. As shown in Figure 1a,b, Ca^2+^ and PO_4_^3−^ will gradually diffuse into the Alg networks. Due to the strong ability of sodium alginate to capture calcium ions, it provides large number of nucleating sites for the nucleation of calcium phosphate [22,23]. Subsequently, in situ mineralization of Dox into calcium phosphate clusters was achieved as shown in Figure 1c. Those clusters would gradually aggregate and grow into Dox@CaP-Alg NSs (greyish spheres at right side in Figure 1d). The products were isolated from solution by centrifugation and then re-suspended for further tea polyphenol functional step. Finally, tea polyphenol functionalized calcium phosphate nanospheres were obtained by a modified tea polyphenol polymerization method according to our previous work and the related literatures [12,24]. The detailed procedures are as followings: 5 mg Dox@CaP-Alg NSs, 27 mg green tea polyphenols (TP) powder, 7.5 mg CaCl_2_ and 50 mL deionized water were added into a 100 mL flask under stirring to form homogeneous solution. After altering its pH to 7.3, the solution was heated for 3 h at 60 °C under reflux. Then, it was natural cooling and kept still for at least three days at ambient temperature. The product was separated from the solution by centrifugation (5000 g/min, 5 min), and rinsed by deionized water for several times. After air-dried, the precipitates (DCAT NSs) were further characterized and analysis.

### 3.4. Drug Loading and Release Determination

The calibration curve of Dox was determined by taking absorbance (480 nm, characteristic adsorption of Dox) versus Dox concentration between 0 and 1 × 10^−4^ mol·L^−1^ as parameters. For this interval, the calibration curve fits the Lambert and Beer’s law:

(1) A=10740.26×C−0.00865

where *A* is the absorbance and *C* is the concentration (mol·L^−1^).

The release of Dox from DCAT NSs was monitored at different pH values (PBS, Ph = 7.4, 0.1 mol·L^−1^; acetate buffer solution, pH = 5.0, 0.1 mol·L^−1^) in the presence (0.5, 1, 2, 3, and 5 mM) and absence of glutathione at 37 °C. At specified time points, the nanospheres were centrifuged and supernatant was taken for UV-vis analysis. Drug loading efficiency (DLE, 93%) and drug loading content (DLC, 21 wt.%) were calculated according to our former work [21].

### 3.5. Cellular Uptake and Cell Viability

The cellular uptake, release and cell apoptosis behaviors of DCAT NSs were investigated by CLSM and 5-dimethylthiazol-2yl-2,5-diphenyl-tetra zolium bromide (MTT) assay. Cells were cultured in Dulbecco’s modified Eagle’s medium (DMEM) supplemented with 10% fetal bovine serum (FBS). For MTT, cells were seeded in a 96-well plate (5 × 10^3^ cells/well) and incubated with various concentrations of DCAT NSs at given pH or GSH concentration for certain time. Cell viability was calculate investigated by measuring light absorbance (OD value) at 490 nm using an ELISA plate reader. When examined by CLSM, the nuclei were stained by Hoechst 33342 and the cytoskeleton was stained by tubulin. MG63 cells and MC3T3 cells were used to evaluate the cell apoptosis in the absence or presence of Dox loaded NSs and free Dox. MG63 or MC3T3 cells were incubated for 24 h at 37 °C in a humidified 5% CO_2_ atmosphere. Subsequently, the cells were treated with free medium with different concentrations of Dox (0.01, 0.1, 1.0, 3, 5, and 10 μg/mL). Then, the plate was incubated for 48 h, at pH = 7.4 or 5.0, respectively.

## 4. Conclusions

We propose a DDS strategy that involves encapsulation of Dox by pH and GSH dual responsive carriers. The obtained Dox encapsulated DCAT NSs having a narrow size range of ~260 nm. The drug loading/release studies reveal that these DCAT NSs have a drug loading efficiency (DLE) of 93% and drug loading content (DLC) of 21 wt.%. Besides, the release mechanism studies of Dox from DCAT NSs were both pH and GSH depended. CLSM images revealed that these DCAT NSs could internalize the cells. DCAT NSs have tumor cell killing effects similar to Dox, however, the side effects of toxicities in normal cells are much lower than Dox. Based on confocal fluorescence microscopy, cytotoxicity, and release mechanism experiments, the prepared tea polyphenol functionalized, doxorubicin loaded, acidity and GSH dual responsive DCAT NSs were confirmed to have highly efficient internalization and obvious cytotoxic effect on target tumor cells, but not normal cells. The effect of variable DOX or TP content, variable dimensions of the nanospheres on the function the obtained DDS will be further investigated in future work.

## Supplementary Materials

The following are available online. Figure S1: the calibration curve of Dox determined by taking absorbance versus Dox concentration between 0 and 1 × 10^−4^ mol·L^−1^ as parameters. Molar absorption coefficient of Dox was calculated as 10,253.6 L·mol^−1^· cm^−1^ (average value). Figure S2: Chemical formula of main components of green tea polyphenols (mainly catechins). Figure S3: Chemical formula of doxorubicin.
