# Supplementary material for: Dialysis Preparation of Smart Redox and Acidity Dual Responsive Tea Polyphenol Functionalized Calcium Phosphate Nanospheres as Anticancer Drug Carriers
Source: Molecules. 2020 Mar 9;25(5):1221. doi: 10.3390/molecules25051221 (PMC7179473; doi:10.3390/molecules25051221)
Supplement: Supplementary file 1 [file molecules-25-01221-s001.pdf]

Supplementary Materials

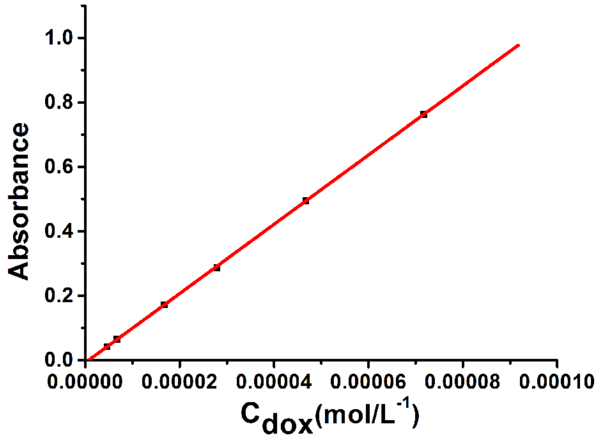

Figure S1. The calibration curve of Dox determined by taking absorbance versus Dox concentration between 0 and 1 × 10^{-4} mol·L^{-1} as parameters. Molar absorption coefficient of Dox was calculated as 10,253.6 L·mol^{-1}·cm^{-1} (average value).

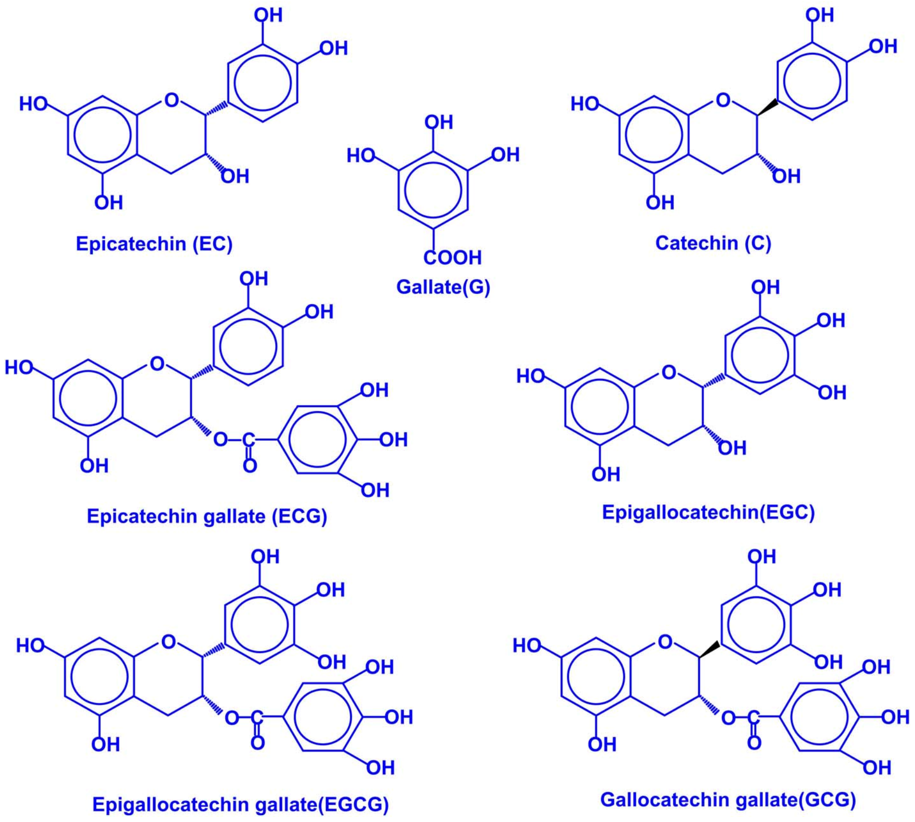

Figure S2. Chemical formula of main components of green tea polyphenols (mainly catechins).

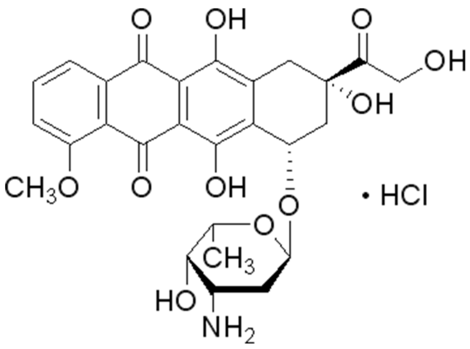

Figure S3. Chemical formula of doxorubicin.
